# Supplementary material for: Symptomatic and Asymptomatic Protist Infections in Hospital Inpatients in Southwestern China
Source: Pathogens. 2021 May 31;10(6):684. doi: 10.3390/pathogens10060684 (PMC8226927; doi:10.3390/pathogens10060684)
Supplement: Supplementary file 1 [file pathogens-10-00684-s001.zip › pathogens-1181145-supplementary.pdf]

Table S1 Oligonucleotides and PCR conditions used for the molecular identification and/or characterization of the intestinal protist parasites investigated in Tengchong City, southwest China.

Enteric pathogen	Locus	Primers name	Primer (5′–3′)	Amplicon size (bp)	Cycling conditions	Reference
Giardia duodenalis	*tpi*	AL3543	AAATIATGCCTGCTCGTCG	605	Denaturation: 94 °C for 45 s	[49]
		AL3546	CAAACCTTITCCGCAAACC		Annealing: 50 °C for 45 s	
		AL3544	CCCTTCATCGGIGGTAACTT	530	Elongation: 72 °C for 1 min	
		AL3545	GTGGCCACCACICCCGTGCC		No. cycles: 35	
Cryptosporidium spp.	*ssu*-rRNA	Primer F1	TTCTAGAGCTAATACATGCG	1,325	Denaturation: 94 °C for 1 min	[48]
		Primer R1	CCCATTTCCTTCGAAACAGGA		Annealing: 55 (1st) or 58 (2nd) °C for 1 min	
		Primer F2	GGAAGGGTTGTATTTATTAGATAAAG	840	Elongation: 72 °C for 1 min	
		Primer R2	CTCATAAGGTGCTGAAGGAGTA		No. cycles: 35	
E. histolytica	*ssu*-rRNA	E1	TGCTGTGATTAAAACGCT	1,947	Denaturation: 94 °C for 1 min	[50]
		E2	TTAACTATTTCAATCTCGG		Annealing: 47 (1st) or 58 (2nd) °C for 1 min	
		Eh-L	ACATTTTGAAGACTTTATGTAAGTA	427	Elongation: 72 °C for 1 min	
		Eh-R	CAGATCTAGAAACAATGCTTCTCT		No. cycles: 35	
Blastocystis sp.	*ssu*-rRNA	Forward	GGAGGTAGTGACAATAAATC	1,100	Denaturation: 94 °C for 1 min	[47]
					Annealing: 54 °C for 1 min	
		Reverse	ACTAGGAATTCCTCGTTCATG		Elongation: 72 °C for 1 min	
					No. cycles: 35	

ssu rRNA, small subunit ribosomal RNA; *tpi*, Triose phosphate isomerase
